# Osteosarcoma Cell-Derived Exosomal miR-1307 Promotes Tumorgenesis via Targeting AGAP1

**DOI:** 10.1155/2021/7358153

**Published:** 2021-03-25

**Authors:** Fei Han, Peidong Pu, Chao Wang, Xiao Ding, Zhoujun Zhu, Wei Xiang, Weishan Wang

**Affiliations:** ^1^Department of Orthopaedics, The First Affiliated Hospital of the Medical College, Shihezi University, Shihezi, China; ^2^Medical College, Shihezi University, Shihezi, China

## Abstract

The occurrence of osteosarcoma (OS) is associated with abnormal expression of many microRNAs (miRNAs). Exosomal miRNAs get much more attentions in intracellular communications. miR-1307 has been studied in many cancers, but its effects in OS have not been studied. We hypothesized that OS-derived exosomal miR-1307 regulates OS tumorigenesis. First, we found OS cell-derived exosomes (Exos) significantly promoted the proliferation, migration, and invasion of OS cells. Secondly, we found miR-1307 was highly expressed in OS cell-derived exosomes (OS-Exos), human OS tissues, and OS cell lines. Then, OS-Exos were extracted after OS cells were cultured and transfected with miR-1307 inhibitor, and the level of miR-1307 in OS-Exos was significantly reduced. When the level of miR-1307 in OS-Exos was significantly reduced, the effects of OS-Exos on migration, invasion, and proliferation of OS cells were also significantly weakened. Furthermore, using TargetScan, miRDB, and mirDIP databases, we identified that AGAP1 was a target gene of miR-1307. Overexpression of miR-1307 could inhibit the expression of AGAP1 gene. We also found AGAP1 was lower expressed in human OS tissues and OS cell lines. Luciferase gene indicated that miR-1307 directly bound the 3'-UTR of AGAP1. miR-1307 was negatively correlated with AGAP1 in clinical study. miR-1307 could significantly promote the proliferation, migration, and invasion of OS cells. In addition, upregulation of AGAP1 could significantly inhibit the role of miR-1307 in OS. In conclusion, our study suggests that OS cell-derived exosomal miR-1307 promotes the proliferation, migration, and invasion of OS cells via targeting AGAP1, and miR-1307-AGAP1 axis may play an important role in the future treatment of OS.

## 1. Introduction

OS is a malignant tumor that usually occurs in adolescents [1], and the incidence is about 0.0004% per year worldwide [2]. The common complications of OS are lung metastases and pathological fractures [3], and the occurrence of lung metastasis will make the patient's prognosis worse [4]. Typical symptoms include pain, limited joint movement, and localized swelling. Although the life of patients has been prolonged after relevant treatment, their physical and mental health has been seriously affected [5]. The occurrence and development of OS is complicated, and many genetic and environmental factors are involved in these processes [6]. Therefore, it is necessary to study the basic molecular mechanisms and understand its pathogenesis of OS for optimizing therapeutic strategies.

More and more studies have shown that miRNAs as a carcinogen or tumor suppressor play an important role in the occurrence and development of tumor [7]. miRNA is a very small RNA molecule that inhibits its expression by directly targeting the 3'-UTR of mRNA [8]. miRNAs can regulate biological activities such as proliferation, invasion, migration, and apoptosis of cancer cells [9]. So far, a number of studies have shown that the abnormal expression of some miRNAs are closely related to the development of OS. For example, miR-488 inhibits apoptosis and promotes proliferation and migration of OS cells via targeting BIM [10], and miR-603 promotes proliferation and invasion of OS cells via targeting BRCC2 [11].

Several studies have shown that miR-1307 promotes the development of tumor. For example, Qiu and Dou found that miR-1307 promoted the proliferation of prostate cancer via targeting FOXO3A [12]. Han et al. found miR-1307 promoted the growth of breast carcinoma via targeting SMYD4 [13]. Chen et al. found that miR-1307 promoted the metastasis of hepatocellular carcinoma via targeting DAB2, and high levels of miR-1307 suggested poor clinical outcomes in patients [14]. And miR-1307 also could promote the proliferation and inhibit the apoptosis of ovarian cancer cells via targeting ING5 [15]. Clinical studies had found that combination of miRNAs including miR-1307, miR-6861-5p, miR-4634, miR-1246, and miR-6875-5p could be used to detect early breast cancer [16]. Thus, miR-1307 may be an oncogene that plays an important role in the development of tumors. Studies have shown that miR-1307 has been used in clinical diagnosis of early breast cancer and the clinical prognosis of liver cancer, indicating that its potential research value and prospect in clinical application are very high. However, the role of miR-1307 in OS and its basic molecular mechanisms are unknown.

Exos are vesicles released by membrane fusion, with a size of 30-150 nm [17]. Exos play an important role in intercellular communication, and studies have shown that siRNA and miRNA can be transferred by Exos [18, 19]. Studies have found that Exos from tumor can promote the growth of tumor [20]. There have been several reports about the relationship between OS and Exos. For example, Shimbo et al. found that mesenchymal stem cell-derived exosomal miR-143 could inhibit the migration of OS cells [21]. Gong et al. found that OS-derived exosomal miR-675 could promote the invasiveness of hFOB1.19 osteoblasts via targeting CALN1 [22]. In addition, carcinoma-associated fibroblasts derived exosomal miR-1228 promote the migration and invasion of OS cells via targeting the SCAI [23]. However, the effects of OS cell-derived Exos and the miRNAs in Exos on OS cells are still unknown; therefore, further research is needed.

In this paper, we found that OS cell-derived exosomal miR-1307 promoted the proliferation, migration, and invasion of OS cells. In addition, we also found that miR-1307 regulated these effects via targeting AGAP1.

## 2. Materials and Methods

### 2.1. Tissue Samples from Clinical Patients

This study was approved by the ethics committee of the First Affiliated Hospital of the Medical College, Shihezi University. The institutional approval number for humans studies was 2017-115-01. OS tissues and adjacent normal bone tissues were collected from 18 patients during surgery diagnosed with OS between 2017 and 2019 (Table S1). Among the 18 patients, 8 were female and 10 were male. All patients without any medical treatment prior to admission signed the written informed consent before registration. There was no anticancer treatment before surgery of all patients in this study.

### 2.2. Cell Culture and Selection of Cell Lines

U2OS and 143B OS cell lines and hFOB1.19 osteoblasts cell line were purchased from ATCC (USA). OS cells were cultured in RPMI-1640 (Hyclone, USA), and hFOB1.19 osteoblasts were cultured in DMEM/F12 (GIBCO, USA) supplemented with 10% FBS (Hyclone, USA), 1% streptomycin, and penicillin maintained at 37°C and in 5% CO_2_.

### 2.3. Isolation and Identification of Exos

U2OS OS cells, 143B OS cells, and hFOB1.19 osteoblasts were cultured in RPMI-1640 or DMEM/F12 supplemented with 10% Exos-free FBS (SBI, USA), 1% streptomycin, and penicillin for 72 h. The medium was collected and centrifuged by differential centrifugation (300 g x 10 min, 2000 g x 10 min, 10000 g x 30 min, and 100000 g x 70 min). The entire process of centrifugation was performed at 4°C, and the samples were stored at -80°C after centrifugation. Exos on copper wire mesh were dried with filter paper and then stain for 1 min. We used transmission electron microscope (TEM) to observe the characteristics of Exos. Nanoparticle tracking analysis (NTA) was used to assay the distribution and size of Exos. hFOB1.19-Exos, U2OS-Exos, or 143B-Exos were cocultured with hFOB1.19 cells, U2OS cells, or 143B cells in DMEM/F12 (GIBCO, USA) or RPMI-1640 (Hyclone, USA) supplemented with 10% FBS (Hyclone, USA), 1% streptomycin, and penicillin maintained at 37°C and in 5% CO_2_.

### 2.4. Exos Labeling and Uptake

Dil (Sigma, USA) as red fluorescence was used to label Exos. First, 2 *μ*g Exos were stained with Dil at room temperature for 15 min. Second, PBS was used to wash off Dil from Exos that has not been stained with ultracentrifuged at 15000 g for 15 min. 2 *μ*g labeled Exos was added into 24-well plates at a density of 9 x 10^3^ hFOB1.19 cells, U2OS cells, or 143B cells cultured in DMEM/F12 (GIBCO, USA) or RPMI-1640 (Hyclone, USA) supplemented with 10% FBS (Hyclone, USA), 1% streptomycin, and penicillin maintained at 37°C and in 5% CO_2_ for 12 h. After incubation for 12 h, the cells were washed three times with PBS and fixed with 4% paraformaldehyde for 30 min. The cell nuclei were labeled with DAPI (Vectorlabs, USA) and then imaged with fluorescence microscope.

### 2.5. Cell Transfection

miR-1307 mimics, miR-1307 inhibitor, miR-NC mimics, and miR-NC inhibitor were purchased from RiboBio (Guangzhou, China). Liquid A and liquid B were prepared, respectively: liquid A: 3 *μ*l mimics or inhibitor was added to 100 *μ*l Opti-MEM (GIBCO, USA) and mixed for 5 min; liquid B: 6 *μ*l Lipofectamine RNAiMAX (Invitrogen, USA) was added to 100 *μ*l Opti-MEM and mixed for 5 min. Then, liquid A and liquid B were mixed for 5 min, and a 209 *μ*l mixture was added to the control group or the experimental group. When the cell density was about 80%, 2 × 10^5^/ml cells were taken and transfected for 48 h. Gene sequences are as follows (5′-3′): miR-1307 mimics, F: ACUCGGCGUGGCGUCGGUCGUG, R: CGACCGACGCCACGCCGAGUUU. miR-1307 inhibitor, F: CACGACCGACGCCACGCCGAGU.

### 2.6. CCK-8 Assay for Cell Proliferation

After the cells were digested, the number of cells were counted, and the concentration of cells were adjusted to 1 x 10^5^ cells/ml. Cells were divided into 96-well plate with 1 x 10^4^ cells/well. hFOB1.19 cells, U2OS cells, or 143B cells were treated with 25 *μ*g/ml hFOB1.19-Exos, U2OS-Exos, or 143B-Exos for 24 h, 48 h, and 72 h. The CCK-8 kit was used to assay the proliferation of OS cells (Promega, USA). After incubation for 4 h, the microplate reader was used to read the absorbance at 450 nm (Thermo Fisher Scientific, USA).

### 2.7. Transwell Assay for Cell Invasion and Migration

Transwell cell culture plates (BD, USA), 50 mg/L Matrigel gels (BD, USA), and chambers with pore size 8 *μ*m (Millicell, Germany) were used. hFOB1.19 cells, U2OS cells, or 143B cells were treated with 25 *μ*g/ml hFOB1.19-Exos, U2OS-Exos, or 143B-Exos for 24 h, 48 h, and 72 h. Serum-free medium and the dissolved gels were mixed in a ratio of 1 : 3, and a 40 *μ*l mixture was added to the precooled Transwell chamber (invasion). Migration experiments did not need Matrigel gels. 1 x 10^5^ cells were resuspended with 100 *μ*l serum-free medium and added to the upper chamber. 600 *μ*l serum medium supplemented with 10% FBS was added to the chamber and incubated at 37°C in 5% CO_2_ for 12-15 hours. They were fixed with 4% paraformaldehyde for 10 min and stained with crystal violet for 20 min, then washed with PBS thrice. The cells were wiped off with cotton in the chamber. The cells were observed whether they passed through the pores under the microscope, and photos were taken with 5 random fields using the ImageJ software for statistics (×20 magnification).

### 2.8. Total RNA Extraction and qRT-PCR Experimental Methods

Collected cells and 1 ml TRIzol (Invitrogen, USA) were used to fully lyse cells. TaqMan®miRNAReverse Transcription Kit was used to prepare the reaction system (ABI, USA). Primers are as follows: GAPDH, U6, AGAP1, and miR-1307 (RiboBio, China). Primers sequences (5′-3′) are as follows: GAPDH, F: GCTCATTTGCAGGGGGGAG, R: GTTGGTGGTGCAGGAGGCA; U6, F: CTCGCTTCGGCAGCACA, R: AACGCTTCACGAATTTGCGT; AGAP1, F: TACGGGCTGAATGTGGA, R: GAGGAATGGCTGGGAGA; miR-1307, F: ACTCGGCGTGGCGTCG, R: TCCTCTCCTCCTTCCTCTTC. 2^-*ΔΔ*CT^ was used to calculate genes' relative expression.

### 2.9. Western Blot

HRP-labeled GAPDH has high-quality internal reference (KC-5G5, Shanghai Kangcheng Biological, China), anti-AGAP1 (ab199136, Abcam, UK), anti-CD9 (ab223052, Abcam, UK), anti-CD63 (ab68418, Abcam, UK), and rabbit anti-Calnexin (ab10286, Abcam, UK). Anti-IgG (ab4030-05, Southern Biotech, USA) was used as the secondary antibody. The secondary antibody was diluted (1 : 20000). 96-well plate was prepared, and 20 *μ*l samples were added to the 96-well plate. Each hole was added with 200 *μ*l liquid, incubated at 37°C, and avoided light for 30 min.

### 2.10. Luciferase Gene

First, PGLO-AGAP1-WT and PGLO-AGAP1-MUT were constructed by RiboBio (Guangzhou, China). miR-1307 mimics, miR-NC mimics, and miR-1307 inhibitor cotransfected with PGLO-AGAP1-WT or PGLO-AGAP1-MUT in OS cells. A dual fluorescence detector (Promega, USA) was used to assay the activity of luciferase.

### 2.11. Statistical Analysis

Data were presented as mean ± SD. Student's *t*-test counted the difference between the two groups. One-way ANOVA was used to perform comparison of multiple groups. *P* < 0.05 indicated statistically significant.

## 3. Results

### 3.1. Identification of Exosomes

**Figure 1:**
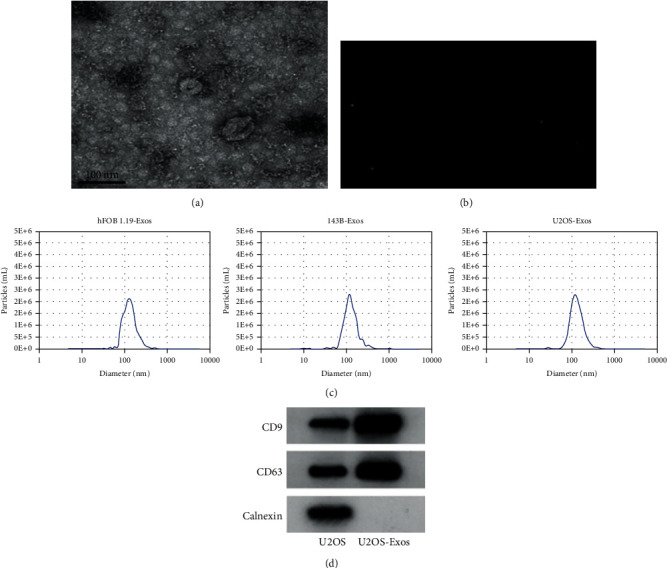
Authentication of OS-Exos. (a) TEM image of U2OS-Exos (scale bars, 100 nm). (b) The moving image of the detected particles. (c) The distribution and size of hFOB1.19-Exos, 143B-Exos, and U2OS-Exos were assayed by NTA. (d) Western blot analysis was used to confirm the absence of Exos-free protein (Calnexin) and the presence of Exos proteins (CD9 and CD63).

U2OS OS cell-, 143B OS cell-, and hFOB1.19 osteoblast-derived particles were isolated from the supernatant by ultracentrifugation. TEM showed that the shape of U2OS-derived particles was round vesicles with bilayer membrane (Figure 1(a)).  Figure 1(b) shows the moving image of the detected particles. NTA showed the diameters of hFOB1.19-, 143B-, and U2OS-derived particles with a size of 30-150 nm (Figure 1(c)). Western blot analysis showed that specific proteins of Exos (CD9 and CD63) were highly expressed in U2OS-Exos (Figure 1(d)). The above data indicate that the extracellular particles isolated in this study are OS-Exos.

### 3.2. OS-Exos Promoted the Proliferation, Migration, and Invasion of OS Cells

**Figure 2:**
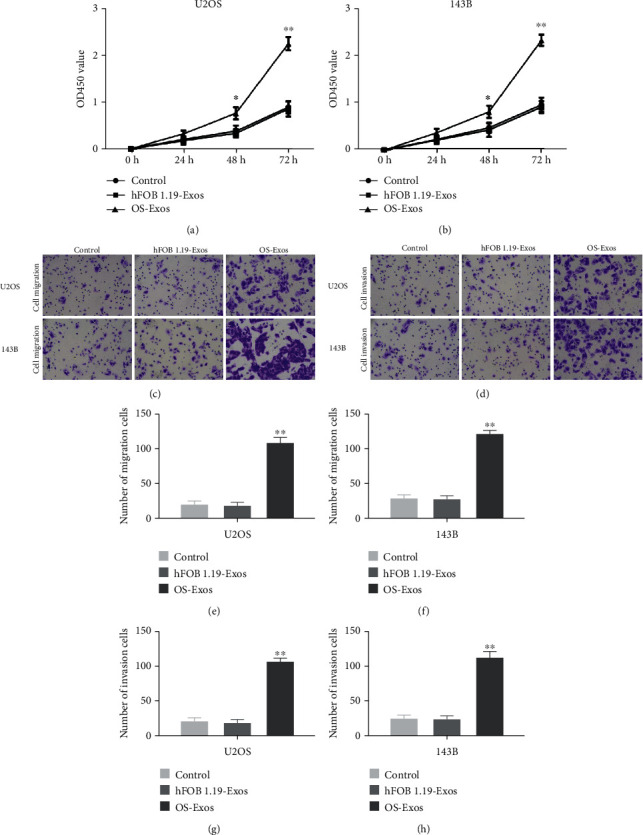
Effects of OS-Exos on proliferation, migration, and invasion of OS cells. (a, b) Effects on proliferation of OS cells after OS cells treated with 25 *μ*g/ml OS-Exos or 25 *μ*g/ml hFOB1.19-Exos were assayed by CCK-8, without Exos treatment as control. (c–h) Effects on migration and invasion of OS cells after OS cells treated with 25 *μ*g/ml OS-Exos or 25 *μ*g/ml hFOB1.19-Exos were assayed by Transwell, without Exos treatment as control. Scale bars, 100 um. ∗*P* < 0.05, ∗∗*P* < 0.01.

Next, we assayed the effects of OS-Exos on proliferation, migration, and invasion of OS cells. After OS cells treated with 25 *μ*g/ml OS-Exos or hFOB1.19-Exos for 24 h, 48 h, and 72 h, we found that OS-Exos significantly promoted the proliferation of OS cells compared with control or hFOB1.19-Exos assayed by CCK-8 (Figures 2(a) and 2(b)). Transwell was used to assay the migration and invasion of OS cells treated with 25 *μ*g/ml OS-Exos or hFOB1.19-Exos for 12-15 h. We found that OS-Exos significantly promoted the migration and invasion of OS cells compared with control or hFOB1.19-Exos (Figures 2(c)–2(g)). These data suggest that OS-Exos promote the proliferation, migration, and invasion of OS cells in a paracrine manner.

### 3.3. Uptake of Exos

**Figure 3:**
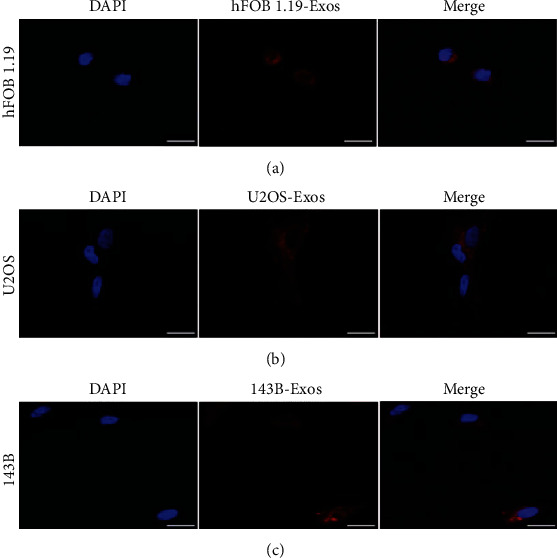
Uptake of Exos by hFOB1.19, U2OS, and 143B. (a–c) Exos were labeled with red fluorescence and lipophilic dye Dil and cocultured with hFOB1.19, U2OS, or 143B for 12 h. Blue fluorescence shows nuclei stained with DAPI. Scale bars, 10 *μ*m.

To determine whether hFOB1.19-, U2OS-, or 143B-derived Exos could be taken up in hFOB1.19, U2OS, or 143B, hFOB1.19-, U2OS-, or 143B-derived Exos were labeled with Dil and cocultured with hFOB1.19, U2OS, or 143B for 12 h (Figures 3(a)–3(c)). Fluorescence microscope showed the Exos were around the nucleus and the morphology of the nucleus did not change. The results show that hFOB1.19-, U2OS-, or 143B-derived Exos could be taken up in hFOB1.19, U2OS, or 143B.

### 3.4. miR-1307 Was Highly Expressed in OS-Exos, OS Cell Lines, and Human OS Tissues

**Figure 4:**
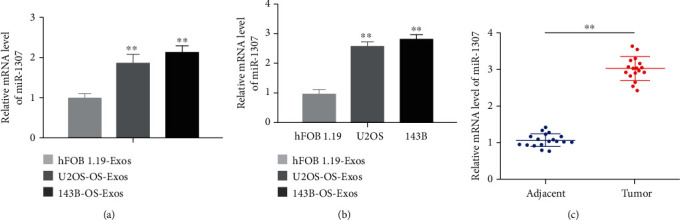
The mRNA levels of miR-1307 in OS-Exos, OS cell lines, 18 paired human OS tissues, and adjacent normal tissues. (a) The mRNA levels of miR-1307 in hFOB1.19-Exos, U2OS-Exos, and 143B-Exos were assayed by qRT-PCR. (b) The mRNA levels of miR-1307 in two OS cell lines and hFOB1.19 cell line were assayed by qRT-PCR. (c) The mRNA levels of miR-1307 in 18 paired human OS tissues and adjacent normal tissues were assayed by qRT-PCR. ∗∗*P* < 0.01.

qRT-PCR was used to analyze the expression levels of miR-1307 in OS-Exos, OS cell lines, 18 paired human OS tissues, and adjacent normal tissues. We found the expression levels of miR-1307 were highly expressed in OS-Exos compared with hFOB1.19-Exos (Figure 4(a)). Also, the expression levels of miR-1307 were highly expressed in two OS cell lines and human OS tissues compared with hFOB1.19 osteoblasts and adjacent normal tissues (Figures 4(b) and 4(c)). To further investigate the relationship between miR-1307 level and clinicopathological features in OS, we divided the median level of miR-1307 in OS tissues as the cut-off point into the low group (<median, *n* = 7) and the high group (>median, *n* = 11). Clinical data showed that the expression level of miR-1307 was positively correlated with the size of OS and the level of serum AKP (Table 1). The results show that miR-1307 can be transferred by OS-Exos and may regulate the function of OS cells.

### 3.5. miR-1307 Mimics or miR-1307 Inhibitor Was Successfully Transfected into OS Cells

**Figure 5:**
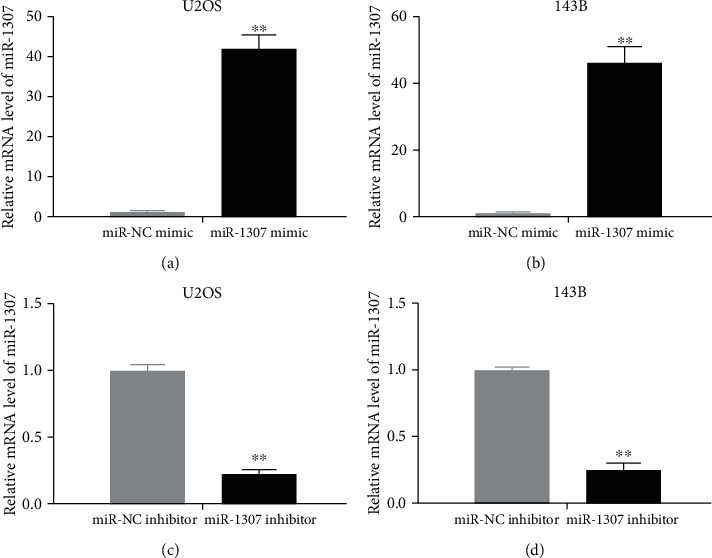
Testing of transfection efficiency. (a, b) The mRNA levels of miR-1307 after OS cells transfected with miR-NC mimics or miR-1307 mimics were assayed by qRT-PCR. (c, d) The mRNA levels of miR-1307 after OS cells transfected with miR-NC inhibitor or miR-1307 inhibitor were assayed by qRT-PCR. ∗∗*P* < 0.01.

First, miR-1307 mimics or miR-1307 inhibitor was transfected into OS cells to confirm the transfection efficiency by qRT-PCR. We found that the mRNA levels of miR-1307 were obviously increased or reduced after OS cells transfected with miR-1307 mimics or miR-1307 inhibitor compared with negative control (Figures 5(a)–5(d)). The above data indicate that miR-1307 mimics or miR-1307 inhibitor can be successfully transfected into OS cells.

### 3.6. OS Cell-Derived Exosomal miR-1307 Promoted the Proliferation, Migration, and Invasion of OS Cells

**Figure 6:**
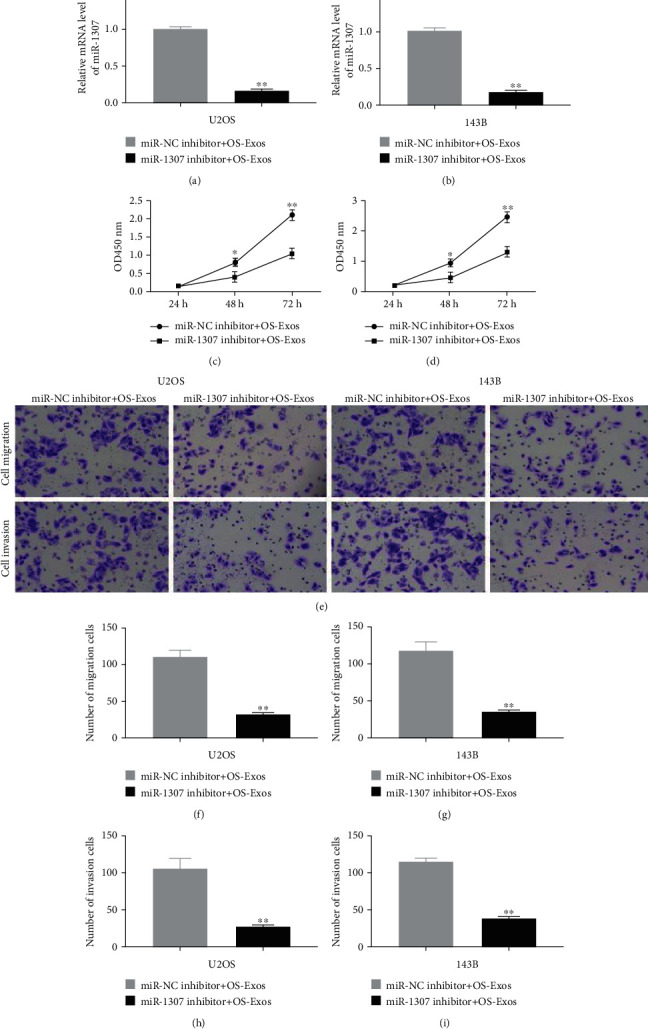
Effects of OS-Exos on proliferation, migration and invasion of OS cells after downregulating miR-1307 in OS-Exos. (a, b) The mRNA levels of miR-1307 in OS-Exos after OS cells transfected with miR-NC inhibitor or miR-1307 inhibitor were assayed by qRT-PCR. (c, d) Effects on proliferation of OS cells after OS cells treated with 25 *μ*g/ml miR-NC inhibitor+OS-Exos or 25 *μ*g/ml miR-1307 inhibitor+OS-Exos were assayed by CCK-8. (e–i) Effects on migration and invasion of OS cells after OS cells treated with 25 *μ*g/ml miR-NC inhibitor+OS-Exos or 25 *μ*g/ml miR-1307 inhibitor+OS-Exos were assayed by Transwell. Scale bars, 100 *μ*m. ∗*P* < 0.05, ∗∗*P* < 0.01.

In order to further investigate whether the regulation of OS-Exos on OS cells was related to miR-1307, we first cultured OS cells and transfected with miR-1307 inhibitor or miR-NC inhibitor and then isolated Exos from OS cells after 72 h. The level of miR-1307 in OS-Exos was obviously reduced after OS cells transfected with miR-1307 inhibitor compared with negative control (Figures 6(a) and 6(b)). CCK-8 was used to assay the proliferation of OS cells. Interestingly, after OS cells treated with 25 *μ*g/ml miR-1307 inhibitor+OS-Exos for 24 h, 48 h, and 72 h, the effect of OS-Exos on proliferation of OS cells was significantly weakened after the level of miR-1307 in OS-Exos was obviously reduced (Figures 6(c) and 6(d)). Similarly, the migration and invasion abilities of OS cells treated with 25 *μ*g/ml miR-1307 inhibitor+OS-Exos for 12-15 h were both reduced compared with negative control as assayed by Transwell (Figures 6(e)–6(i)). These data show that OS cell-derived exosomal miR-1307 promotes the proliferation, migration, and invasion of OS cells.

### 3.7. AGAP1 Was a Target Gene of miR-1307

**Figure 7:**
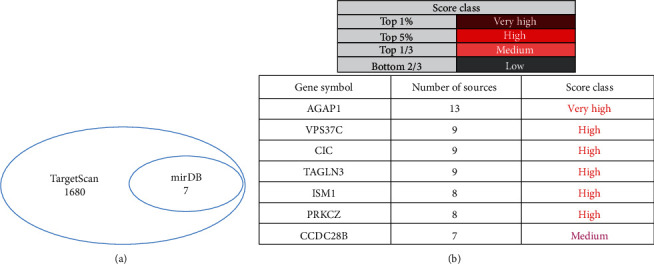
TargetScan, mirDIP, and miRDB databases were used to predict the target genes of miR-1307. (a) miRDB and TargetScan databases were used to select the intersection of predicted target genes of miR-1307. (b) mirDIP database showed the comprehensive binding scores between AGAP1 gene and miR-1307.

TargetScan, mirDIP, and miRDB databases were used to predict the target genes of miR-1307. First, the intersection of predicted target genes (7 target genes) was selected by miRDB and TargetScan databases (Figure 7(a)). Then, the mirDIP database was used to compare the comprehensive binding scores between 7 target genes and miR-1307; among the predicted target genes, only the score classification (very high) of AGAP1 was the highest (Figure 7(b)). So, we chose AGAP1 for further study.

### 3.8. miR-1307 Inhibited the Expression of AGAP1 Gene, and the Expression Levels of AGAP1 Were Lower Expressed in OS Cell Lines and Human OS Tissues

**Figure 8:**
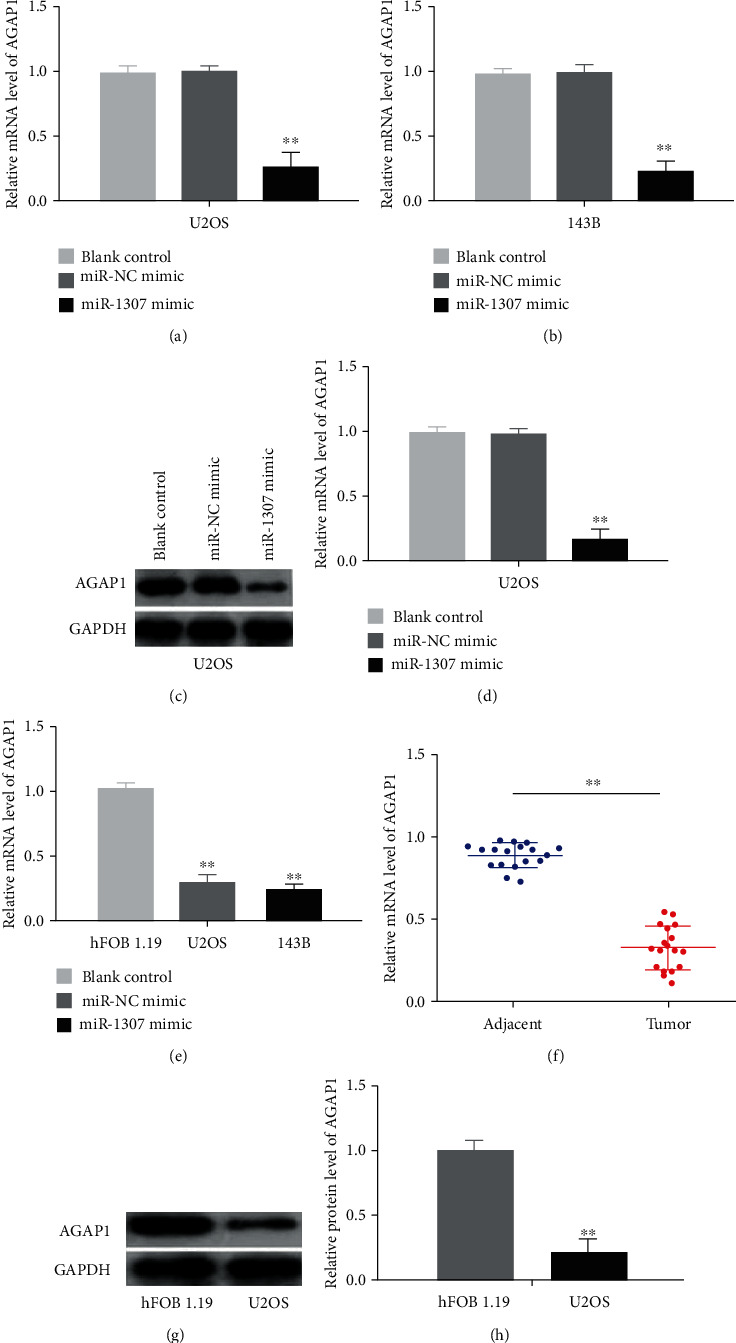
miR-1307 inhibited the expression of AGAP1 gene and the expression levels of AGAP1 in OS cell lines and human OS tissues. (a, b) The mRNA levels of AGAP1 after OS cells transfected with PBS, miR-NC mimics, or miR-1307 mimics were assayed by qRT-PCR. (c) The protein levels of AGAP1 after U2OS cells transfected with PBS, miR-NC mimics, or miR-1307 mimics were assayed by western blot. (d) Relative protein level of AGAP1. (e) The mRNA levels of AGAP1 in two OS cell lines and hFOB1.19 cell line were assayed by qRT-PCR. (f) The mRNA levels of AGAP1 in 18 paired human OS tissues and adjacent normal tissues were assayed by qRT-PCR. (g) The protein level of AGAP1 in hFOB1.19 cells and U2OS OS cells was assayed by western blot. (h) Relative protein level of AGAP1. ∗∗*P* < 0.01.

Our study showed the expression levels of miR-1307 were highly expressed in human OS tissues and OS cell lines, and AGAP1 was a target gene of miR-1307. Therefore, we assayed whether overexpression of miR-1307 could inhibit the expression of AGAP1 gene. As assayed by qRT-PCR (Figures 8(a) and 8(b)) and western blot (Figures 8(c) and 8(d)), the mRNA and protein levels of AGAP1 were significantly reduced after OS cells transfected with miR-1307 mimics compared with negative control or blank control. Next, we assayed the expression levels of AGAP1 in human OS tissues and OS cell lines. We found the mRNA levels of AGAP1 were lower expressed in two OS cell lines and human OS tissues compared with hFOB1.19 osteoblasts and adjacent normal tissues (Figures 8(e) and 8(f)). Also, the protein level of AGAP1 was lower expressed in U2OS OS cells compared with hFOB1.19 osteoblasts (Figures 8(g) and 8(h)). To further investigate the relationship between AGAP1 level and clinicopathological features in OS, we divided the median level of AGAP1 in OS tissues as the cut-off point into the low group (<median, *n* = 10) and the high group (>median, *n* = 8). Clinical data showed that the expression level of AGAP1 was negatively correlated with the size of OS and the level of serum AKP (Table 2). The results show that miR-1307 may regulate the growth of OS via targeting AGAP1, and AGAP1 may be a potential therapeutic target in OS treatment.

### 3.9. miR-1307 Directly Targeted the 3'-UTR of AGAP1 in OS Cells

**Figure 9:**
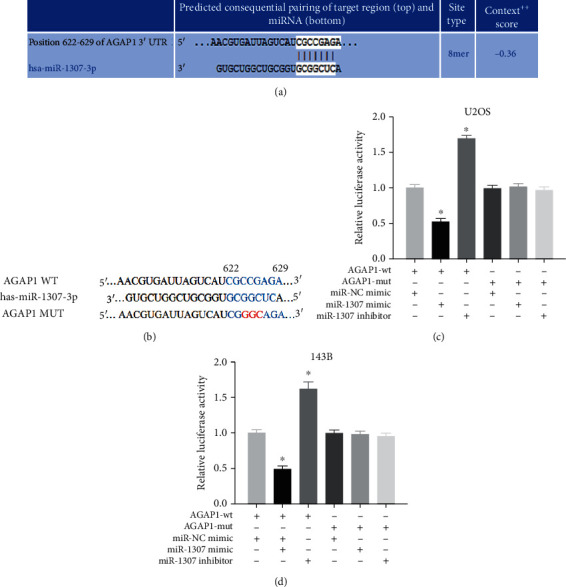
miR-1307 directly targeted the 3'-UTR of AGAP1 in OS cells. (a) The complementary sequence between the 3'-UTR of AGAP1 and miR-1307. (b) The wild 3'-UTR of AGAP1 (WT) and mutant 3'-UTR of AGAP1 (MUT) were designed and mutated sites were labeled with red areas. (c, d) Luciferase technology is used to assay the luciferase activity after OS cells transfected with miR-NC mimics, miR-1307 mimics, or miR-1307 inhibitor. ∗*P* < 0.05.

To further verify whether miR-1307 directly targeted the 3'-UTR of AGAP1, first, the TargetScan database was used to find a complementary sequence between the 3'-UTR of AGAP1 and miR-1307 (Figure 9(a)). Second, the wild 3'-UTR of AGAP1 (WT) and mutant 3'-UTR of AGAP1 (MUT) were designed to assay the activity of luciferase (Figure 9(b)). Luciferase activity was significantly decreased after miR-1307 cotransfected with AGAP1 WT PGLO-3'-UTR vector in OS cells, but not in MUT. Similarly, luciferase activity was significantly increased after miR-1307 inhibitor cotransfected with AGAP1 WT PGLO-3'-UTR vector in OS cells, but not in MUT (Figures 9(c) and 9(d)). The above data indicate that miR-1307 directly targets the 3'-UTR of AGAP1.

### 3.10. miR-1307 Promoted the Proliferation, Migration, and Invasion of OS Cells via Targeting AGAP1

**Figure 10:**
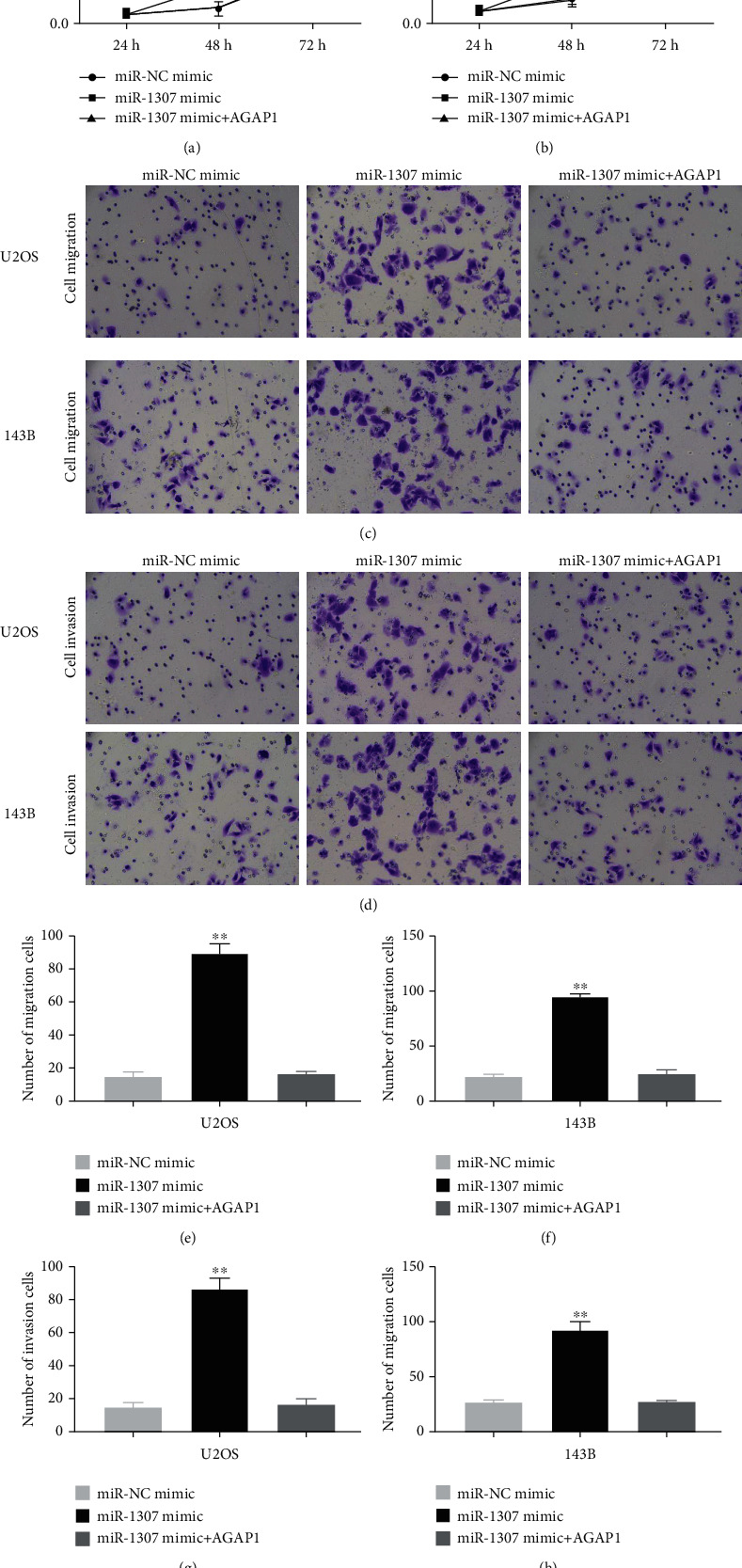
miR-1307 regulated the growth of OS cells via targeting AGAP1. (a, b) Effects on proliferation of OS cells after OS cells transfected with miR-NC mimics, miR-1307 mimics, or miR-1307 mimics+AGAP1 protein. (c–h) Effects on migration and invasion of OS cells after OS cells transfected with miR-NC mimics, miR-1307 mimics, or miR-1307 mimics+AGAP1 protein. Scale bars, 100 *μ*m. ∗*P* < 0.05, ∗∗*P* < 0.01.

To further understand whether miR-1307 regulated the proliferation, migration, and invasion of OS cells via targeting AGAP1, OS cells were treated with miR-NC mimics, miR-1307 mimics, or miR-1307 mimics+AGAP1. We found that miR-1307 obviously promoted the proliferation of OS cells compared with miR-NC, whereas, when added to AGAP1 protein, miR-1307 failed to promote the proliferation of OS cells (Figures 10(a) and 10(b)). Consistently, we also found that miR-1307 obviously promoted the migration and invasion of OS cells compared with miR-NC. However, these effects were weakened when added to AGAP1 protein (Figures 10(c)–10(h)). These data show that miR-1307 promotes the proliferation, migration, and invasion of OS cells, at least, partially via targeting AGAP1.

## 4. Discussion

miRNAs are reported to be involved in the occurrence and development of tumor as a carcinogen or tumor suppressor [24]. Recent studies have revealed many miRNAs play important roles in the development of OS. For example, miRNA-134 was shown to inhibit the proliferation, invasion, and metastasis and to promote the apoptosis of OS cells [25]. Taheriazam et al. found that the expression of miRNA-218 was downregulated, and the expression of miRNA-130b was upregulated in OS tissues [26]. Creighton et al. found that overexpression of miRNA-31 inhibited the proliferation of OS cells [27]. However, our study found the effects of miR-1307 and the basic molecular mechanisms in OS.

In this study, we first extracted OS cell- and hFOB1.19 osteoblast-derived Exos, then, respectively, added them to OS cells. The results showed that OS-Exos significantly promoted the proliferation, migration, and invasion of OS cells. Fluorescence microscope showed that hFOB1.19-, U2OS-, or 143B-derived Exos could be taken up in hFOB1.19, U2OS, or 143B. Second, we found that the expression levels of miR-1307 were highly expressed in OS-Exos, OS cell lines, and human OS tissues. In order to further investigate whether the regulation of OS-Exos on OS cells was related to miR-1307, we reduced the expression level of miR-1307 in OS-Exos. When OS cells transfected with miR-1307 inhibitor, the level of miR-1307 was obviously reduced in OS cells. Meanwhile, the level of miR-1307 was also obviously reduced in OS-Exos. Interestingly, the effects of OS-Exos on proliferation, migration, and invasion of OS cells were significantly weakened after the level of miR-1307 in OS-Exos was obviously reduced. TargetScan, mirDIP, and miRDB databases were used to predict the target genes of miR-1307, and we found AGAP1 was a target gene of miR-1307. Next, we assayed whether overexpression of miR-1307 could inhibit the expression of AGAP1 gene. The mRNA and protein levels of AGAP1 were significantly reduced after OS cells transfected with miR-1307 mimic. Importantly, we found the mRNA levels of AGAP1 were lower expressed in human OS tissues and two OS cell lines, and the protein level of AGAP1 was also lower expressed in U2OS OS cells. Luciferase gene showed that miR-1307 directly targeted the 3'-UTR of AGAP1, thus inhibiting the mRNA and protein level of AGAP1. To further understand whether miR-1307 regulated the proliferation, migration, and invasion of OS cells via targeting AGAP1, OS cells were treated with miR-NC mimics, miR-1307 mimics, or miR-1307 mimics+AGAP1 protein. Finally, miR-1307 could significantly promote the proliferation, migration, and invasion of OS cells, while overexpression of AGAP1 could inhibit the above regulatory effects of miR-1307. In summary, OS cell-derived exosomal miR-1307 promotes the proliferation, migration, and invasion of OS cells at least partially via targeting AGAP1, which may provide new molecular target and therapeutic strategy for the treatment of OS.

Many studies had shown that tumor-derived exosomes were involved in the positive regulation of tumor. For example, nasopharyngeal carcinoma-derived exosomal miR-17-5p promoted angiogenesis of nasopharyngeal carcinoma via targeting BAMBI [28]. Ovarian cancer-derived exosomal miR-205 could promote the development of ovarian cancer [29]. Pancreatic cancer-derived exosomal miR-27a promoted the development of pancreatic cancer via targeting BTG2 [30]. Tumor-derived Exos could deliver MyD88, Hsp72, MICA∗008, HER2, miR-223, and let-7 miRNA family to promote the development of tumor through regulating angiogenesis and cell growth of tumor [31–33]. Thus, tumor-derived Exos can be used as a carrier of miRNA to regulate the growth and metabolism of tumor cells. Although the effects of OS-Exos on proliferation, migration, and invasion of OS cells are significantly weakened after the level of miR-1307 in OS-Exos is obviously reduced, it still has a little influence on OS cells. So, we cannot exclude possibilities that other miRNAs or small molecules within the Exos also participate in regulating the proliferation, migration, and invasion of OS cells.

In this study, we found AGAP1 as a direct target gene of miR-1307 mediated the effects of Exos in regulating OS cells. AGAP1 was an Arf GAP that depended phosphoinositide, and it had some effects on actin and endocytic compartment [34]. Studies show that AGAP1 was a novel binding partner of NO sensitive guanylyl cyclase [35]. Bendor et al. found that AGAP1 regulated endocytic recycling of M5 and promoted dopamine release [36]. Tsutsumi et al. shown that AGAP1 regulated FilGAP and inhibited the invasion of breast cancer and glioma cells [37]. Although our study indicates that AGAP1 mediates the effects of miR-1307 on proliferation, migration, and invasion of OS cells, other target genes of miR-1307 may exist which also contribute to these effects of miR-1307.

Studies had shown that AKP levels in patients with OS were significantly increased and positively correlated with osteogenic activity [38]. Clinical data showed that the expression level of miR-1307 was positively correlated with the size of OS and the level of serum AKP, and the expression level of AGAP1 was negatively correlated with the size of OS and the level of serum AKP. The results suggest that the levels of miR-1307 and AGAP1 in OS tissues reflect the size of OS and the level of serum AKP, which may provide some value for the diagnosis and treatment of OS.

In this study, the effects of OS cell-derived Exos on OS cells were compensated to some extent. Although we assayed the expression levels of miR-1307 and AGAP1 in OS cell lines and human OS tissues, we did not verify that OS cell-derived exosomal miR-1307 regulated the proliferation, migration, and invasion of OS cells in animal model. We realize that there are a huge gap and differences between in vitro study and in vivo study. In future study, we will test whether OS-Exos or exosomal miR-1307 could regulate the growth, development, angiogenesis, and metastasis of OS cells in animal models.

In conclusion, our study demonstrated that OS-Exos significantly promoted the proliferation, migration, and invasion of OS cells. miR-1307 was highly expressed in OS cells, OS-Exos, and human OS tissues. When miR-1307 was downregulated in OS-Exos, the effects of OS-Exos on proliferation, migration, and invasion of OS cells were significantly reduced. miR-1307 directly bound the 3'-UTR of AGAP1, thereby inhibiting the mRNA and protein levels of AGAP1. We also found AGAP1 was significantly underexpressed in OS cells, and miR-1307 was negatively correlated with AGAP1 in clinical study. miR-1307 could significantly promote the proliferation, migration, and invasion of OS cells, while overexpression of AGAP1 could inhibit the role of miR-1307 in OS, which could be used as a potential therapeutic target for OS. In summary, our study indicated that OS cell-derived exosomal miR-1307 promotes the proliferation, migration, and invasion of OS cells via targeting AGAP1.

## Figures and Tables

**Table 1:** Association between expression of miR-1307 and clinical patients.

miR-1307				
Parameters	*N*	High group (*n* = 11)	Low group (*n* = 7)	*P*
Age (years)				
<18	7	4	3	0.783
≥18	11	7	4	
Gender				
Male	10	6	4	0.914
Female	8	5	3	
Tumor location				
Leg	15	10	5	0.280
Others	3	1	2	
Tumor size (cm)				
<5	10	4	6	0.040∗
≥5	8	7	1	
Enneking stage				
IA/IB	3	2	1	0.829
IIB/III	15	9	6	
Histologic type				
Conventional	10	5	5	0.280
Others	8	6	2	
Pulmonary metastasis				
Yes	5	4	1	0.308
No	13	7	6	
Pathological fracture				
Yes	2	1	1	0.732
No	16	10	6	
Serum AKP				
Normal	6	1	5	0.006∗
Increase	12	10	2	
KPS score				
≥80	13	7	6	0.308
≤70	5	4	1	

Chi-square test, ∗*P* < 0.05.

**Table 2:** Association between expression of AGAP1 and clinical patients.

AGAP1				
Parameters	*N*	High group (*n* = 8)	Low group (*n* = 10)	*P*
Age (years)				
<18	7	3	4	0.914
≥18	11	5	6	
Gender				
Male	10	5	5	0.596
Female	8	3	5	
Tumor location				
Leg	15	6	9	0.396
Others	3	2	1	
Tumor size (cm)				
<5	10	7	3	0.015∗
≥5	8	1	7	
Enneking stage				
IA/IB	3	1	2	0.671
IIB/III	15	7	8	
Histologic type				
Conventional	10	3	7	0.168
Others	8	5	3	
Pulmonary metastasis				
Yes	5	2	3	0.814
No	13	6	7	
Pathological fracture				
Yes	2	1	1	0.867
No	16	7	9	
Serum AKP				
Normal	6	5	1	0.019∗
Increase	12	3	9	
KPS score				
≥80	13	6	7	0.814
≤70	5	2	3	

Chi-square test, ∗*P* < 0.05.

## Data Availability

The data used to support the findings of this study are available from the corresponding author upon request.

## Supplementary Materials

Supplementary Materials Table S1: detailed clinical data of 18 patients with OS involved in this study.
