# Expression of Concern on “Osteosarcoma Cell‐Derived Exosomal miR‐1307 Promotes Tumorgenesis via Targeting AGAP1”

**DOI:** 10.1155/bmri/9783976

**Published:** 2026-06-03

**Authors:** BioMed Research International

EXPRESSION OF CONCERN: F. Han, P. Pu, C. Wang, X. Ding, Z. Zhu, W. Xiang, and W. Wang, “Osteosarcoma Cell‐Derived Exosomal miR‐1307 Promotes Tumorgenesis via Targeting AGAP1”, *BioMed Research International*, vol. 2021 (2021). https://doi.org/10.1155/2021/7358153.

This Expression of Concern is for the above article, published online on 25 March 2021 in Wiley Online Library (wileyonlinelibrary.com), and has been issued following the agreement of the Journal’s Editor‐in‐Chief, Yoel A. Klug; and John Wiley & Sons Ltd.

The Expression of Concern has been issued due to the investigation of the concerns flagged by *Acaenotus canus* on PubPeer [1]. The concerns related to the similarity of the panel showing the cell invasion of 143B cells from the control group in Figure 2d to the panel in Figure 10c, which denoted the results of a cell migration assay of 143B cells treated with miR‐1307 mimic+ AGAP1. The authors were contacted on several occasions to provide the raw data supporting the affected figures; however, the publisher did not receive the raw data requested. In light of this, the journal team could not verify the validity of these images and could not exclude that the above‐mentioned concerns affect the overall conclusions of the article. Therefore, the journal has decided to issue an Expression of Concern to inform and alert readers.
