# Supplementary material for: Osteosarcoma Cell-Derived Exosomal miR-1307 Promotes Tumorgenesis via Targeting AGAP1
Source: Biomed Res Int. 2021 Mar 25;2021:7358153. doi: 10.1155/2021/7358153 (PMC8016573; doi:10.1155/2021/7358153)
Supplement: Supplementary Materials — Table S1: detailed clinical data of 18 patients with OS involved in this study. [file 7358153.f1.pdf]

Table S1 Detailed clinical data of 18 patients with osteosarcoma

N	Age	Gender	Tumor location	Tumor size (cm)	Enneking stage	Histologic type	Pulmonary metastasis	Pathological fracture	Serum AKP levels before treatment	KPS score
1	9	Female	Femur	4.5	Ⅱ B	Osteoblastic	No	No	Increase	80
2	11	Female	Tibia	5.4	Ⅱ B	Fibroblast	No	No	Increase	80
3	11	Male	Femur	4.8	Ⅱ B	Chondroblast	No	No	Increase	80
4	12	Male	Femur	3.7	Ⅱ B	Osteoblastic	No	No	Normal	80
5	14	Female	Tibia	3	Ⅰ B	Well differentiated in bone	No	No	Normal	90
6	15	Female	Femur	9.2	Ⅲ	Dedifferentiat-ed parosteal	Yes	No	Increase	70
7	15	Male	Femur	3.4	Ⅱ B	Chondroblast	No	No	Normal	80
8	18	Male	Humerus	3.3	Ⅰ B	Parosteal	No	No	Normal	90
9	20	Male	Femur	8.8	Ⅲ	Round cell	Yes	No	Increase	70
10	21	Female	Spine	3.8	Ⅱ B	Osteoblastic	No	No	Increase	80
11	24	Male	Tibia	11.6	Ⅲ	Malignant fibrous histiocytoma	Yes	No	Increase	70
12	29	Male	Femur	10.4	Ⅲ	Telangiectatic	Yes	Yes	Increase	60
13	33	Male	Humerus	2.5	Ⅰ A	Parosteal	No	No	Normal	90
14	36	Female	Tibia	4.1	Ⅱ B	Osteoblastic	No	No	Increase	80
15	41	Male	Femur	4.4	Ⅱ B	Fibroblast	No	No	Normal	80
16	48	Female	Tibia	5	Ⅱ B	Osteoblastic	No	No	Increase	80
17	55	Female	Femur	12.3	Ⅲ	Telangiectatic	Yes	Yes	Increase	50
18	61	Male	Femur	5.4	Ⅱ B	Fibroblast	No	No	Increase	80
